# [Retracted] Molecular mechanism of atractylon in the invasion and migration of hepatic cancer cells based on high‑throughput sequencing

**DOI:** 10.3892/mmr.2024.13373

**Published:** 2024-10-21

**Authors:** Yang Cheng, Jian Ping, Jianjie Chen, Yifei Fu, Hui Zhao, Jiahua Xue

Mol Med Rep 25: 112, 2022; DOI: 10.3892/mmr.2022.12628

Following the publication of this paper, it was drawn to the Editor's attention by a concerned reader that certain of the Transwell invasion assay data shown in Fig. 5E on p. 9 were strikingly similar to data that had already been published in different form in another article written by different authors at a different research institute [Zuo K, Zhao Y, Zheng Y, Chen D, Liu X, Du S and Liu Q: Long non-coding RNA XIST promotes malignant behavior of epithelial ovarian cancer. Onco Targets Ther 12: 7261–7267, 2019].

Owing to the fact that the contentious data in the above article had already been published prior to its submission to *Molecular Medicine Reports*, the Editor has decided that this paper should be retracted from the Journal. The authors were asked for an explanation to account for these concerns, but the Editorial Office did not receive a satisfactory reply. The Editor apologizes to the readership for any inconvenience caused.

